# Analysis of the Flux Performance of Different RO/NF Membranes in the Treatment of Agroindustrial Wastewater by Means of the Boundary Flux Theory

**DOI:** 10.3390/membranes9010002

**Published:** 2018-12-26

**Authors:** Javier M. Ochando-Pulido, Antonio Martínez-Férez, Marco Stoller

**Affiliations:** 1 Department of Chemical Engineering, University of Granada, Avenida de la Fuente Nueva S/N C.P., 18071 Granada, Spain; anferez@ugr.es; 2 Department of Industrial Engineering, University of Salerno, Via Giovanni Paolo II, 132, 84084 Fisciano, Italy; marco.stoller@uniroma1.it

**Keywords:** boundary flux, reverse osmosis, nanofiltration, olive mill wastewater, membrane pretreatments, wastewater reclamation

## Abstract

Dynamic membrane system behaviour must be adequately addressed to avoid process unfeasibility. The lack of proper analysis will mean relying on erroneous permeate flux values in the system design, which will lead to quick and/or steady high fouling rates. In this paper, the authors present additional data supporting the boundary flux theory as a helpful tool for membrane engineers to carefully avoid process failures. By fitting the dynamic permeate flux data to the boundary flux model, it was possible to calculate the β fouling index for the three selected membranes (one nanofiltration (NF) and two reverse osmosis (RO) ones). The dynamic flux given by the low-pressure RO membrane did not follow sub-boundary operating conditions, since a sharp flux loss was measured throughout the whole operating cycle, pinpointing that supra-boundary flux conditions were governing the system. This was supported by the calculated value of the β fouling parameter, which resulted to be in the order of ten times higher for this membrane. However, the values of β→0 for the SC-RO and DK-NF ones, supported by the very low value of the sub-boundary fouling parameter α (0.002 and 0.007 L·h^−1^·m^−2^·bar^−2^, respectively), ensure nearly boundary operating conditions for these membranes.

## 1. Introduction

Nanofiltration (NF) and reverse osmosis (RO) membranes are starting to be amply used in many applications nowadays, particularly in wastewater treatment processes, in substitution of conventional separation operations or in an integrated form. This situation is the result of the research and innovation made in new membrane materials, designs, module conceptions and general knowhow during the last decades [1,2]. NF membranes display many advantages for separation and purification purposes, mainly their ability to permit pollutant removal without the need for reagents, providing average product quality regardless of the feedstream characteristics; ability to operate at ambient temperature; compactness; and capability to comply with regulations of water quality standards. Average NF pore sizes range from about 0.1 to 1 nm, thus both high- and low-molecular-weight particles can be rejected, whereas the smallest molecules and ions (mainly monovalent) permeate through the membrane. Moreover, if adequately chosen, NF membranes can operate under moderate pressure whilst yielding relatively high fluxes.

If high levels of purification are pursued, NF is the subsequent membrane to ultrafiltration (UF). As an example, NF membranes are currently employed in the following industrial sectors: pharmaceutical, oil and petroleum, gas purification, production of natural essential oils and similar products, and agrofood industries, as well as in wastewater treatment plants, among others.

On another hand, reverse osmosis (RO) membranes can be used to comply with the most stringent standards. For instance, RO membranes can be found in a vast range of industrial and agroindustrial wastewater treatment plants, such as stainless steel, energy cogeneration, nuclear power, and textile and food industries, among others [3,4,5,6].

In all these processes, fouling limits negatively the technical and economic efficiency. On one hand, the concentration of solutes in the boundary of the membrane triggers concentration polarisation. This establishes an additional resistance to the solvent passage, hence raising the operating costs as a result of flux decrease, reducing the permeate quality due to the increment of concentration gradient across the membrane thickness; that is, causing alteration of the membrane selectivity. Membrane fouling is complex and comprises different possible mechanisms, some of which can take place simultaneously: pore blocking, plugging and clogging, chemical degradation, and/or cake formation. In addition, according to its origin, it is classified in organic, inorganic, or biofouling.

Fouling hinders the membranes’ performances. If fouling is not understood a priori and well controlled, the initial performances will be quickly reduced. Moreover, if the membrane suffers from irreversible pore blockage or scaling, its service lifetime will be shortened, resulting in process design failure [7,8,9,10].

In most situations, apparent critical fluxes may not be sufficient to avoid membrane fouling. This is the case for wastewater treatment [11], for which fouling will trigger unavoidably at a higher or lower extent. In case of working above the critical conditions, exponential fouling buildup can cause high permeate flux reduction rates, in many cases leading to irreversible fouling formation that will make the recovery of the membrane permeability utterly difficult [7,8,11].

In spite of the formulation of different models to describe and fill the knowledge gap of membrane fouling phenomena, a lack of reliability still remains at the industrial scale. Fouling boosts the specific energy consumption needed to reach the permeate flow target, thus increasing the operating costs, also because of the necessary plant shut-downs for membrane cleaning-in-place (CIP). Also, irreversible fouling increases the capital costs because of membrane module substitution.

To address these issues, engineers’ tendency is to use wide safety margins to overdimension the membrane plants to take fouling into account [12,13]. Even if this can maintain the process performance in time, the capital costs will be incremented considerably. In other cases, fouling is underestimated and leads to rapid process failures.

Stoller and Ochando-Pulido introduced the boundary flux concept, which merges critical and threshold flux concepts together into one [12,13]. They highlighted that the boundary flux value of a membrane process depends not only on the formerly identified variables, but also changes dynamically over time. In this manuscript, the boundary flux concept will be used to determine the flux safe operating framework for NF and RO membranes to treat agroindustrial wastewater.

## 2. Materials and Methods

### 2.1. Membrane Bench-Scale Plant Fitted with NF/RO Membranes

Membrane experiments were performed in a bench-scale tangential flow membrane unit (Prozesstechnik GmbH). The flow scheme of the system is fully reported elsewhere [6]. The pretreated olive mill wastewater (OMW) was contained in a double-walled tank (5 L maximum volume), driven to the flat module (3.9 cm width × 33.5 cm length) by a diaphragm pump (Hydra-Cell D-03). The principal operating parameters (net pressure, temperature, tangential velocity) were measured and controlled: (i) the operating pressure was controlled independently from the flowrate, set with a spring-loaded pressure regulating valve, and monitored by a digital pressure gauge, whereas (ii) the system temperature was controlled by an automatic electronic temperature controller.

The characteristics of the used and virgin membranes (NF and RO, GE Water and Process Technologies) are reported in Table 1. The membranes had an active surface of 200 cm^2^. The hydraulic permeability of each membrane was determined by measuring the pure water flux over their range of applied pressures at ambient temperature and turbulent tangential velocity. Before the beginning of the experiment, the membrane was conditioned by filtering MilliQ^®^ water at a fixed pressure to permit the compaction and swelling of the membrane, until a stable flux was measured.

NF and RO experiments (replicated twice) were run in a semicontinuous manner at ambient temperature (22 ± 0.1 °C). Moreover, turbulent tangential flow was set over the membrane (Reynolds number > 4 × 10^3^) to provide the minimisation of concentration polarisation phenomena on the membrane surface. The P_set point_ (±0.01 bar) was set at different values for each membrane, as reported in Table 2. This means, when taking into account a 200-mL dead volume within the system, a volume recovery factor (VRF) of 90%.

The permeate flux was dynamically measured with a precision electronic mass balance (AX-120 Cobos, Mettler, Toledo, Spain, ±0.1 mg).

At the end of every semibatch run, the membrane was subjected to cleaning in situ with 0.1–0.15% *w*/*v* NaOH and 0.1–0.15% *w*/*v* sodium dodecyl sulfate (SDS) solutions (purchased from Panreac S.A., Granada, Spain).

### 2.2. Application of the Boundary Flux Theory for Membrane Performance Analysis and Control

Bacchin et al. and Oringer et al. pointed out local conditions leading to the formation of different liquid/gel phases over the membrane surface and in the pores, as derived from concentration polarisation profiles [9].

Concentration polarisation (CP) is the development of a concentration profile in the fluid phase adjacent to the membrane. Its occurrence in pressure-driven membrane processes is a result of the interplay of convective/diffusive fluxes in the laminar boundary layer adjacent to the membrane. CP is a precursor of fouling and its minimisation should always be considered in the design of NF and RO systems. The quantification of CP in the steady state is given by the film model and considers a mass transfer coefficient that is highly dependent on the cross-flow velocity through the Reynolds number [14]. Thus, the cross-flow velocity is a parameter that plays a major role in concentration polarisation and therefore in fouling minimisation.

Depending on the reversibility, membrane fouling can be: (1) of reversible nature, that is, the sort of fouling which follows the driving force (ΔP_set_) and can be completely removed by flushing or soft cleaning once the pressure is again reduced; (2) semireversible, which cannot be just eliminated easily and needs some cleaning or washing with aqueous solutions of appropriate chemicals; or (3) irreversible, which causes membrane process failure as it cannot be removed from the membrane (i.e., severe scaling, organic or biofouling). During the operation of membrane separation processes, some of these types of fouling will appear to some extent.

An important milestone in the field of membrane fouling knowledge and control was the concept of the critical/threshold flux [7,10]. It is based on the observation that there is an apparent flux value, related to the applied ΔP_set_, above which the fouling rate and irreversibility increases exponentially, and therefore the applied pressure is not controlling anymore. 

Critical and threshold flux equations can be merged, as described by Stoller and Ochando [13]:

dm/dt = −α; J_p_(t) ≤ J_b_ (1)

dm/dt = −α − β (J_p_(t) − J_b_); J_p_(t) > J_b_ (2)

where:

- J_b_ is the boundary flux (L·h^−1^·m^−2^).
- m represents the membrane permeability (L·h^−1^·m^−2^·bar^−1^);
- J_p_ is the permeate flux at any time (L·h^−1^·m^−2^).
- α is the sub-boundary fouling rate index, which indicates the constant permeability reduction rate of the membrane (L·h^−2^·m^−2^·bar^−1^).
- β is the supra-boundary fouling rate index (h^−1^·bar^−1^), determining the fouling pattern in the exponential fouling regime of the membrane system. β is a function of the applied transmembrane pressure (P_TM_) respect to the P_TMb_:

β (P_TM_) = ζ (P_TM_ − P_TMb_) (3)

where ζ is a dimensional fitting parameter and P_TMb_ (bar) is the applied transmembrane pressure corresponding to the boundary flux.

Besides this, the model can be completed with equations on mass balances and on rejection, as detailed elsewhere [13].

Within sub-boundary conditions (Equation (1)), the prediction of the permeate flux at a certain time J_p_*(t) can be performed from the initial permeate flux J_p_(t_1_) once the α index is calculated [13]:

(4) $$J_{p}{}^{*}\left(t\right)\;={\;J}_{p}\left(t_{1}\right)-\alpha \int P_{\mathrm{TM}}\;\mathrm{dt}$$

If Equation (1) fits the dynamic performance of the membrane well, this situation will indicate that merely low (reversible) fouling is being built up (this will help maximise the membrane’s service lifetime). In this regard, operation below J_b_ can ensure long-term yield. In addition to this, α will determine the working period cycle of the membrane before having to stop for CIP. Provided the value of the α fouling index is minimised, the need for CIP—which implies operation stop and costs—will be reduced.

The method to estimate the boundary flux is adapted from the critical flux measurement [15], such that the experimental data serve for the following set of equations (Equations (5)–(7)) to define the two operation ranges; that is, to calculate the values of α and β.

If the membrane permeability reduction in a certain period stays below the boundary conditions, its integration will lead to:

m (t’) − m(t) = Δm* = −α·(t’ − t)(5)

where the dynamic permeability of the membrane is a function of:

m(t) = J_p_(t)/P_TM_(t) (6)

Hence, the boundary flux can be integrated between times t and t’ to determine −∆J_b_*:

(7) $$J_{b}(P_{\mathrm{TM}},\;t)\;-\;J_{b}(P_{\mathrm{TM}},\;t^{\prime })\;=\;-\Delta J_{b}*\;=\;-\int_{t}^{t^{\prime }}\alpha \cdot P_{\mathrm{TM}}\cdot \mathrm{dt}$$

This would permit the rewriting of Equations (1) and (2) as:

(8) $$J_{p}\left(t\right)={\;J}_{p}\left(t_{1}\right)-{\;\alpha \;P}_{\mathrm{TM}}\;\left(t-t_{1}\right)\;;{\;J}_{p}\left(t\right)\leq {\;J}_{b}\left(t\right)$$

(9) $$J_{p}\left(t\right)={\;J}_{p}\left(t_{1}\right)-\;\frac{\alpha -{\;\beta \;J}_{p}\left(t_{1}\right)+{\;\beta \;J}_{b}}{\beta }\;\left(e^{-{\beta \;P}_{\mathrm{TM}}\;\left(t-{\;t}_{1}\right)}-1\right);{\;J}_{p}\left(t\right)>{\;J}_{b}\left(t\right)$$

It can be seen that with the above boundary flux conditions, the additional impact of term β will lead to an exponential decrement of the membrane flux. This means that additional increments of the P_TM_ beyond P_TMb_ will not be conducive to higher stable fluxes. By verifying the goodness of the fitting of Equations (5)–(9) to the membrane system, it would be possible to effectively differentiate into two operating ranges and therefore determine the boundary conditions, rather more simply than by measuring the exponential flux loss in long-term experiments.

### 2.3. Analytical Methods

Analyses of the chemical oxygen demand (COD), total suspended solids (TSS), electrical conductivity (EC), and pH were performed following standard methods [16]. The methods were triplicated and performed with analytical-grade reagents (99% minimum purity).

A Helios Gamma UV–visible spectrophotometer (Thermo Fisher Scientific, Granada, Spain) was used for the analyses of the COD. EC and pH were analysed with a Crison GLP31 conductivity meter and a Crison GLP21 pH meter. TSS were analysed following a standard filtration method [16].

Also, the effluent was characterised according to the saturation index (SI), determined following the ASTM International method [17].

## 3. Results and Discussion

Beforehand, the key features (pH, electrical conductivity (EC), total suspended solids (TSS), chemical oxygen demand (COD), and solubility index (SI)) of the used feedstock were analysed and are reported in Table 3. The feedstream was olive mill wastewater from modern two-phase olive-oil extraction factories (OMW-2), pretreated by coagulation and an advanced oxidation process (Fenton) as described elsewhere [18,19]. The effluent is characterised by a slightly basic pH and a high EC and remaining organic matter (COD) after the pretreatment. Also, the value of the SI, which indicates the propensity of the effluent to trigger the formation of precipitates on the membrane layer, warns about the possibility of scaling.

Subsequently, the permeability of the selected membranes with pure water (m_0_, L·h^−1^·m^−2^) and OMW-2 were measured. Results are given in Table 4.

The membranes’ water permeability (m_0_, L·h^−1^·m^−2^·bar^−1^) was measured in the lab, and resulted as 1.4 for SC and 6.1 for AK (RO membranes) and 8.2 for DK (NF membrane). On another hand, considerably low values were yielded with OMW-2 as the feed (m_OMW-2_, L·h^−1^·m^−2^): 0.89 for SC and 3.9 for AK (RO membranes) and 4.2 for DK (NF membrane); this means a reduction of the permeability of the used membranes equal to 48.8%, 36.1%, and 35.7%, respectively. Similar membrane permeability reduction was experimented by both RO membranes (35.7–36.1%), but was higher in the case of the NF membrane (48.8%), which may be attributed to the existence of pores in this membrane (mean pore diameter D_p_ = 0.5 nm, see Table 1), which are additionally prone to be fouled, in contrast with the dense RO membrane surfaces.

**Figure 1:**
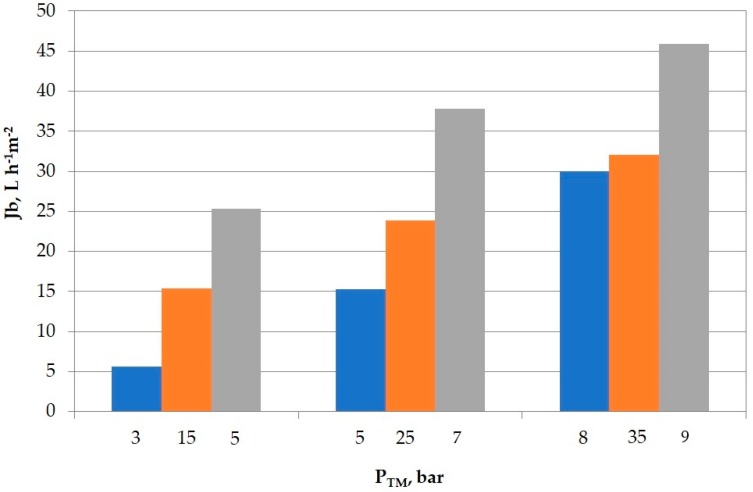
Boundary flux (J_b_, L·h^−1^·m^−2^) measured as a function of the applied transmembrane pressure (P_TM_, bar) for the three membranes tested: RO-AK (blue), RO-SC (orange), and NF-DK (grey). Operating conditions: 22 °C, 5.1 m/s, volume recovery factor (VRF) ≥ 90%.

In Figure 1, the boundary flux values (J_b_, L·h^−1^·m^−2^) measured for the range of each membrane’s P_TM_ (bar) for the three membranes tested are given.

A sensibly higher P_TM_ is required upon operating with the SC-RO membrane, made of composite polyamide/polysulfone, to attain flux values as high as the ones provided by the low-pressure RO membrane (AK), fabricated of asymmetric aromatic polyamide, or the NF-DK one. At P_TM_ of 35 bar, a flux of 32.1 L·h^−1^·m^−2^ was produced by the SC-RO membrane, but merely at 8 bar, 30.0 L·h^−1^·m^−2^ was obtained with the AK-RO membrane and 25.3 L·h^−1^·m^−2^ upon just 5 bar with the DK-NF membrane (Table 5).

When the permeate productivity of both RO membranes (low-pressure AK vs composite SC) were compared, high values were observed for the first one, in contrast with the latter. AK is capable of yielding major fluxes at lower operating pressures, thus a priori, it may seem to be an optimal RO membrane in terms of process feasibility (operating costs’ optimisation).

However, this information is not sufficient and may lead to system design and control failures. One of the principal problems among the available information published relies on the fact that the relation between the operating conditions and the membranes’ performance is mostly disregarded or simply not addressed. An optimised operating framework of a given membrane is, in most cases, the key to succeed in the objectives of production and selectivity goals. In this sense, the use of the boundary flux theory can be a reliable optimisation tool for membrane plant engineers, not only for the dimensioning of the plant, but also to control process failures.

There is a counterbalance that requires attention between operating at high permeate fluxes, which leads to higher fouling, but lower capital costs, and operating upon minor fluxes, which increments capital costs, but can help maintain fouling under control. Moreover, operation design and control of batch membrane processes imply an additional difficulty, which is the variability of the feed as the volume recovery increases. The current work is focused on the examination of batch processes.

**Figure 2:**
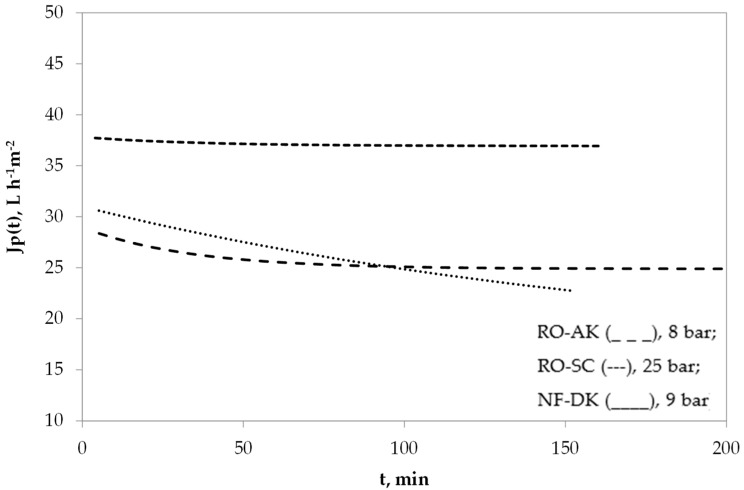
Experimental vs boundary-flux-modelled dynamic permeate flux (J_p_(t), L·h^−1^·m^−2^) of the three membranes tested. Operating conditions: 22 °C, 5.1 m/s, VRF ≥ 90%.

As shown in Figure 2, even though the maximum permeate flux offered by the AK-RO membrane is above that of the SC-RO one at much lower pressure, the analysis of the dynamic operation of the membrane is of key importance, since it reveals that the flux yielded by the former membrane quickly decreases, due to concentration polarisation and fouling phenomena being more critical for this membrane. In fact, this can be explained by the much rougher surface of the AK membrane in contrast with the SC one [20].

The correct design and operation control of a membrane plant is highly affected by the ability of engineers to predict the fouling phenomena that will take place in the system, in which the characteristics of the feed (wastewater in this case) and the membrane are interlinked. As observed by different authors [11,12,13,14,15,16,17,18,19], the determination of the flux point range (J_b_) that ensures operations stay away from high-fouling conditions is needed to enable the continuous and stable operation of the process. Otherwise, frequent stops for maintenance will be necessary to recover the membrane, and the productivity will be affected. These conditions are specific for each feed and membrane system.

As can be seen in Figure 2, the dynamic flux yielded by the low-pressure RO membrane (AK) does not follow the sub-boundary operating conditions, since a sharp permeate flux loss was attained throughout the entire operating period, indicating that supra-boundary flux conditions are being developed within the system for this RO membrane.

In fact, by fitting the dynamic permeate flux data to the set of equations reported for the boundary flux, it was possible to determine the β fouling index for the three selected membranes. This is supported by the calculated value of the β fouling parameter, which resulted to be in the order of ten times higher for the low-pressure membrane.

However, for the other two membranes (SC-RO and DK-NF), the β fouling parameter was estimated to be equal to 0.79 × 10^−6^ and 0.76 × 10^−6^, respectively; that is, β→0, indicating nearly boundary operating conditions. This is confirmed by the plateau observed in the dynamic flux at the steady state for these two membranes (Figure 2).

On another hand, the rejection values were not highly affected during operation, and in the case of the RO membranes, could be maintained at very high values (above 98%). Therefore, this had little impact in attaining the standard values required for the treated effluent. To sum up, TSS could be completely rejected by all three membranes, whereas the COD and EC rejection values were, respectively: 98.8 ± 0.2% and 98.5 ± 0.2% for SC, 98.9 ± 0.2% and 99.2 ± 0.1% for AK, vs 54.0 ± 0.3% and 30 ± 0.5% for DK, as per the conditions stated in Table 6.

In addition to this, the hydraulic permeabilities of the membranes after the cleaning process revealed the complete recovery of the composite RO membrane (SC) and the NF one, but the impossibility of restoring the initial permeability of the asymmetric RO membrane (AK), which showed 20.3% loss, hence denoting irreversible fouling.

These results are interesting, since they raise again the question underlined by Le Clech and coworkers, who reported some cases in which it was not possible to ensure zero fouling rates [11]. It becomes again evident that the interactions of membranes with complex effluents differ from those with single suspensions, such as the ones used to sustain the critical flux concept [7,8], concretely in case of RO membrane systems.

On the other hand, it results in the patent fact that the adequate analysis of the dynamic membrane system behaviour is key for the adequate design of membrane processes, which is a lack detected by the authors in many papers and research studies [13]. If this is not addressed, it will mean relying on erroneous permeate flux values in the system design, which will not be achieved or will lead to quick and/or continuous high fouling rates, making the process unfeasible. In this regard, the boundary flux theory can provide membrane designers with a tool to avoid process failures.

Finally, the value of the sub-boundary fouling parameter was calculated for the SC-RO and DK-NF membranes. Results are reported in Table 7.

The value of the α parameter determines how long the membrane can work continuously without operation shut-downs for cleaning procedures, which represent a certainly undesirable cost and pausing of operation. Therefore, low α-value membranes should be pursued instead of high α-value ones. The values of β→0 for the SC-RO and DK-NF ones, supported by the very low value of the sub-boundary fouling parameter α (0.002 and 0.007 L·h^−1^·m^−2^·bar^−2^, respectively), ensure nearly boundary operating conditions for these membranes.

## 4. Conclusions

The boundary flux theory can provide membrane designers with a helpful tool to carefully avoid process failures. The adequate analysis of the dynamic membrane system behaviour is key for the adequate design of membrane processes, which is a lack detected by the authors in many papers and research studies to date. If this is not addressed, it will mean relying on erroneous permeate flux values in the system design, which will not be achieved or will lead to quick and/or continuous high fouling rates, making the process unfeasible.

The dynamic flux yielded by the low-pressure RO membrane (AK) does not follow the sub-boundary operating conditions, since a sharp permeate flux loss was attained throughout the entire operating period, indicating that supra-boundary flux conditions are being developed within the system for this RO membrane.

By fitting the dynamic permeate flux data to the set of equations reported for the boundary flux, it was possible to determine the β fouling index for the three selected membranes. This is supported by the calculated value of the β fouling parameter, which resulted to be in the order of ten times higher for the low-pressure membrane.

However, for the other two membranes (SC-RO and DK-NF), the β fouling parameter was estimated to be equal to 0.79 × 10^−6^ and 0.76 × 10^−6^, respectively. This is confirmed by the plateau observed in the dynamic flux at the steady state for these two membranes. For these two membranes, a value of the sub-boundary fouling parameter (α, L·h^−1^·m^−2^·bar^−2^), representing the constant permeability loss of the membranes during operation, equal to 0.002 and 0.007, respectively, was finally estimated. The values of β→0 for the SC-RO and DK-NF ones, supported by the very low value of the sub-boundary fouling parameter α (0.002 and 0.007 L·h^−1^·m^−2^·bar^−2^, respectively), ensure nearly boundary operating conditions for these membranes.

## Figures and Tables

**Table 1:** Membranes’ specifications.

Membrane Type	Membrane Model	Material	Structure	Surface Property	Pore Size (nm)	MWCO (Da)	Max. P (bar)	Max. T (°C)
NF	DK	PA */PS **	TFC ^+^	Hydrophilic	0.5	50–300	32	90
RO	AK	Aromatic PA *	Asym. ^+^	Hydrophilic	<0.1	-	9	50
RO	SC	PA */PS **	TFC ^++^	Hydrophilic	<0.1	-	40	90

NF: nanofiltration; RO: reverse osmosis; MWCO: molecular weight cut off; P: pressure; T: temperature; * PA: polyamide; ** PS: polysulfone; ^+^ Asym.: asymmetric; ^++^ TFC: thin-film composite.

**Table 2:** Operating pressure values set on each membrane.

Membrane Type	Membrane Model	P (bar)
NF	DK	5, 7, 9
RO	AK	3, 5, 8
RO	SC	15, 20, 25

Operating conditions: 22 ± 0.1 °C and Reynolds number (Re) > 4 × 10^3^.

**Table 3:** Key physicochemical characteristics of the feedstream measured.

Parameter (Units)	Value
pH	6.9–7.4
EC, mg·L^−1^	3.4–3.6
TSS, mg·L^−1^	13.5–80.9
COD, mg·L^−1^	150.8–290.2
SI	0.44–0.50

EC: electrical conductivity; TSS: total suspended solids; COD: chemical oxygen demand; SI: solubility index.

**Table 4:** Measured pure water permeability (m_0_, L·h^−1^·m^−2^·bar^−1^) and permeability with olive mill wastewater OMW-2 (m_OMW-2_) of the virgin membranes used.

Membrane	m_0_, L·h^−1^·m^−2^·bar^−1^	m_OMW-2_, L·h^−1^·m^−2^·bar^−1^
DK (NF)	8.2	4.2
AK (RO)	6.1	3.9
SC (RO)	1.4	0.9

**Table 5:** Permeate flux (J_p_, L·h^−1^·m^−2^) yielded by the used membranes.

Membrane	P_TM_ (bar)	Permeate Flux J_p_ (L·h^−1^·m^−2^)
SC-RO	15	15.4
	25	23.4
	35	32.1
AK-RO	3	5.6
	5	15.2
	8	30.0
DK-NF	5	25.3
	7	37.8
	9	45.9

Operating conditions: 22 °C, 5.1 m/s, VRF ≥ 90%.

**Table 6:** Fouling indexes and boundary flux for semibatch membrane experiments.

Membrane	P_TM_ (bar)	Fouling Index (β), s^−1^ (×10^−6^)	J_b_ (L·h^−1^·m^−2^)	R^2^
SC-RO	25	0.79	24.3	0.990
AK-RO	8	9.0	16.4	0.989
DK-NF	7	0.76	36.9	0.992

Operating conditions: 22 °C, 5.1 m/s, VRF ≥ 90%.

**Table 7:** Sub-boundary fouling parameter (α, L·h^−1^·m^−2^·bar^−2^).

Membrane	P_TM_ (bar)	Sub-Boundary Fouling Parameter (α)(L·h^−1^·m^−2^·bar^−2^)
SC-RO	25	0.002
DK-NF	7	0.007

Operating conditions: 22 °C, 5.1 m/s, VRF ≥ 90%.
